# X-ray structures of the high-affinity copper transporter Ctr1

**DOI:** 10.1038/s41467-019-09376-7

**Published:** 2019-03-27

**Authors:** Feifei Ren, Brandon L. Logeman, Xiaohui Zhang, Yongjian Liu, Dennis J. Thiele, Peng Yuan

**Affiliations:** 1 Department of Cell Biology and Physiology, Washington University School of Medicine, Saint Louis, MO 63110 USA; 2 Center for the Investigation of Membrane Excitability Diseases, Washington University School of Medicine, Saint Louis, MO 63110 USA; 3 Department of Pharmacology and Cancer Biology, Duke University School of Medicine, Durham, NC 27710 USA; 4 Mallinckrodt Institute of Radiology, Washington University School of Medicine, Saint Louis, MO 63110 USA; 5 Department of Biochemistry, Duke University School of Medicine, Durham, NC 27710 USA; 6 Department of Molecular Genetics and Microbiology, Duke University School of Medicine, Durham, NC 27710 USA; 7 Present Address: Department of Molecular and Cellular Biology, Harvard University, 52 Oxford Street, Cambridge, MA 02138 USA

## Abstract

Copper (Cu) is an essential trace element for growth and development and abnormal Cu levels are associated with anemia, metabolic disease and cancer. Evolutionarily conserved from fungi to humans, the high-affinity Cu^+^ transporter Ctr1 is crucial for both dietary Cu uptake and peripheral distribution, yet the mechanisms for selective permeation of potentially toxic Cu^+^ ions across cell membranes are unknown. Here we present X-ray crystal structures of Ctr1 from *Salmo salar* in both Cu^+^-free and Cu^+^-bound states, revealing a homo-trimeric Cu^+^-selective ion channel-like architecture. Two layers of methionine triads form a selectivity filter, coordinating two bound Cu^+^ ions close to the extracellular entrance. These structures, together with Ctr1 functional characterization, provide a high resolution picture to understand Cu^+^ import across cellular membranes and suggest therapeutic opportunities for intervention in diseases characterized by inappropriate Cu accumulation.

## Abstract (web summary)

Copper (Cu) is an essential trace element for growth and development and the Cu^+^ transporter Ctr1 is crucial for both dietary Cu uptake and peripheral distribution. Here authors solve Cu^+^ -free and Cu^+^ -bound Ctr1 structures which adopt a homo-trimeric Cu^+^ -selective ion channel-like architecture

## Introduction

Copper (Cu) functions as both a cellular signaling agent and a critical cofactor for enzymes involved in a wide spectrum of biochemical processes, including mitochondrial respiration, iron mobilization, superoxide detoxification, connective tissue maturation, and hormone and neuropeptide biogenesis^1^. Defective Cu acquisition underlies neurological, cardiac, connective tissue and innate immune disorders and iron-deficiency anemia, while tissue Cu overload is associated with Wilson’s disease, cancer, and neurodegeneration^2,3^. In biology, Cu exists in two oxidation states (reduced Cu^+^ and oxidized Cu^2+^) and this redox activity has been utilized by Cu-dependent enzymes for catalysis^1^. The high-affinity Cu transporter Ctr1 (*K*_*m*_ ≈ 2 μM)^4^, an integral membrane protein localized to the plasma membrane and endosomal vesicles, mediates cellular Cu uptake in eukaryotes from fungi, insects, plants, fish and vertebrates, thus playing a broad but unique role in biology^5,6^. Several lines of evidence indicate that Ctr1 is highly selective for Cu^+^ over Cu^2+^. The yeast Ctr1 activity requires the Fre family of metalloreductases present at the cell surface^7–9^, and treatment with the reducing agent ascorbate enhances Cu uptake in yeast and cultured mammalian cells^4^. Consistently, the isoelectric Ag^+^, often acting as a surrogate for Cu^+^, effectively competes for Ctr1 mediated Cu uptake^4^.

Systemic or tissue-specific Ctr1 deletion experiments in mice demonstrate the essential role for Ctr1 in embryonic development, dietary Cu acquisition and cardiac and liver function^10–14^. Ctr1, together with the structurally unrelated Cu^+^-transporting ATPase pumps ATP7A and ATP7B that localize to intracellular membranes, in which mutations cause Menkes and Wilson’s diseases, respectively^3^, orchestrates Cu absorption, intracellular Cu distribution, and Cu mobilization to the periphery^15^. Moreover, Ctr1 has been implicated in mediating cellular uptake of platinum-based anticancer drugs, such as cisplatin through endocytosis^16,17^, a mechanism distinct from Cu^+^ transport through the ion pathway created by the Ctr1 protein.

As the only known mammalian Cu importer, Ctr1 is a potential pharmacological target for modifying diseases caused by perturbed Cu levels. However, the structure and mode of Cu^+^ permeation by Ctr1, as well as the selectivity of Ctr1 for Cu^+^ versus Cu^2+^ ions, are poorly understood. A low-resolution (~7–15 Å) model of human Ctr1, obtained by two-dimensional electron crystallography, revealed a symmetric trimer architecture^18,19^, corroborating previous biochemical and genetic characterization of membrane topology and trimeric assembly^4,20–22^. To provide a molecular understanding of Cu^+^ conduction by Ctr1, we determine the X-ray crystal structures of an engineered *Salmo salar* Ctr1 protein, revealing an ion channel-like architecture, and unambiguously identify two Cu^+^ binding sites along the permeation pathway using X-ray anomalous diffraction. Together with mutagenesis studies, we establish a framework for understanding the mechanism by which Ctr1 transports Cu^+^ ions across cellular membranes. These studies provide information to guide therapeutic avenues for the treatment of a broad spectrum of diseases associated with disturbed Cu metabolism.

## Results

### Structure determination

**Fig. 1:**
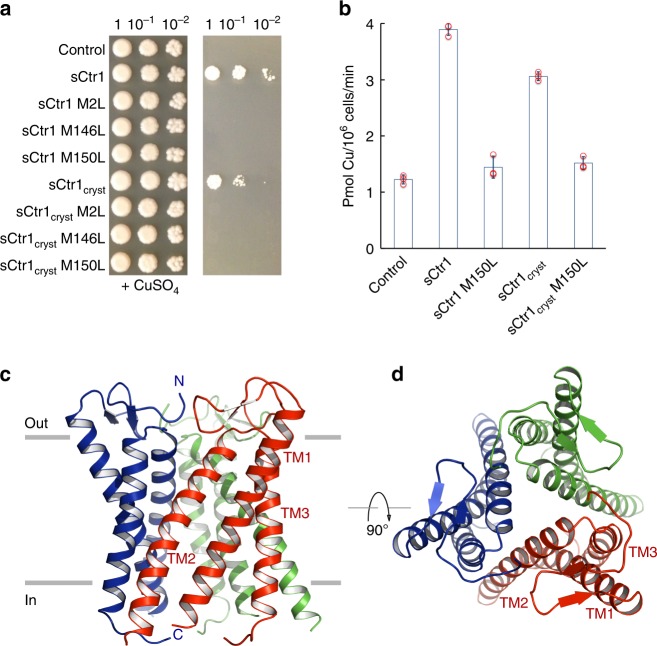
Structure and function of sCtr1. **a** Functional complementation of sCtr1 constructs including the full-length wild-type sCtr1 and inactive mutants sCtr1 M146L, sCtr1 M150L, and sCtr1 M2L (M146L/M150L double mutant) and the corresponding constructs in the sCtr1_cryst_ background. Empty vector is the negative control. Growth on YPEG plates without the supplement of Cu (right panel) requires a functional Cu transporter expressed in the *S. cerevisiae* strain MPY17. Cells were plated as drops with 10-fold serial dilutions. Experimental detail is described in Methods. **b** ^64^Cu uptake by the *S. cerevisiae* strain MPY17 expressing sCtr1 and sCtr1_cryst_ constructs. Values are mean ± s.d. and data were collected from three independent measurements. Source data are provided as a Source Data file. **c** Side view of the symmetric sCtr1 trimer, with each subunit uniquely colored. Gray lines indicate the approximate membrane boundary demarcating outside and inside of the plasma membrane. The N- and C- termini and transmembrane helices are labeled. **d** Top view from the extracellular side

We produced three-dimensional crystals of multiple Ctr1 homologs including human Ctr1 (hCtr1), but the crystals diffracted X-rays to limited resolution, precluding atomic structure determination. After extensive screening and optimization, we improved X-ray diffraction using an engineered *Salmo salar* Ctr1 (78% sequence identity with human Ctr1) construct termed sCtr1_cryst_ (Supplementary Figs. 1 and 2), in which the extracellular N-terminal domain was largely deleted and the intracellular loop connecting TM1 and TM2 was replaced by a crystallization chaperone, the thermo-stabilized apocytochrome b_562_RIL (BRIL)^23^. The crystal construct sCtr1_cryst_ remains functional in Cu uptake, albeit with reduced activity, as assessed by radioactive ^64^Cu uptake experiments and growth assays using an *S. cerevisiae* strain that lacks functional Cu^+^ transporters^4,20,24^ (Fig. 1a, b and Supplementary Fig. 3). The overall Cu uptake rates for cells expressing the wild-type sCtr1 and sCtr1_cryst_ are comparable. However, sCtr1_cryst_ shows much higher expression in the plasma membrane (Fig. 1b and Supplementary Fig. 3).

We determined the crystal structure of sCtr1_cryst_ in the Cu^+^-free state at 3.0 Å resolution (Supplementary Fig. 4 and Table 1). Experimental phases to 3.4 Å resolution were calculated from a single-wavelength anomalous diffraction experiment with a Ta_6_Br_12_-derived crystal. Experimental electron density, shown alongside the final refined 2F_o_−F_c_ electron density (Supplementary Fig. 4), is of sufficient quality for model building. The final sCtr1_cryst_ structural model was refined to 3.0 Å resolution following anisotropic correction (resolution limits of 3.4, 3.5, and 3.0 Å along the reciprocal cell directions a*, b*, and c*, respectively) and was of good quality with respect to a medium resolution (Table 1).

**Table 1:** Data collection, phasing, and refinement statistics for SAD structures

	Cu^+^-free Ctr1 (Ta_6_Br_12_ derivative)	Ctr1-Cu^+^ (Cu edge)	Cu^+^-free Ctr1 (Zn edge)
*Data collection*			
Space group	H32	H32	H32
Cell dimensions			
* a*, *b*, *c* (Å)	a = b = 73.848	a = b = 73.451	a = b = 73.764
	c = 410.577	c = 409.105	c = 408.873
α, β, γ (°)	90, 90, 120	90, 90, 120	90, 90, 120
Wavelength	1.2546	1.3776	1.2820
Resolution (Å)	3.0	3.2	3.5
*R*_sym_ or *R*_merge_	0.121 (1.578)	0.083 (1.178)	0.087 (0.931)
*I* / σ*I*	16.8 (1.3)	21.7 (1.2)	17.2 (1.8)
Completeness (%)	99.3 (99.0)	99.6 (99.7)	99.7 (100)
Redundancy	5.5 (5.4)	6.9 (6.0)	6.0 (6.2)
*Refinement*			
Resolution (Å)			
Low	20	20	
High (a*, b*, c*)^a^	3.4, 3.5, 3.0	3.6, 3.6, 3.2	
No. reflections	6911	5931	
Completeness (%)^b^	77.8	79.9	
*R*_work_ / *R*_free_	0.280 / 0.332	0.301 / 0.340	
No. atoms			
Protein	1622	1560	
Ligand/ion	37	3	
*B*-factors			
Protein	75.93	114.68	
Ligand/ion	95.87	116.89	
R.m.s deviations			
Bond lengths (Å)	0.008	0.009	
Bond angles (°)	1.315	1.152	

*A single crystal was used for each structure determination. *Values in parentheses are for highest-resolution shell. *R*_*free*_ was calculated with 10% of the data

^a^Reflections beyond these limits were excluded from refinement after anisotropic correction (a*, b*, and c* indicate reciprocal cell directions)

^b^Completeness after anisotropic correction

### Trimeric ion channel-like structure

The overall sCtr1_cryst_ symmetric trimer architecture is consistent with genetic evidence for homo-oligomerization and the low-resolution electron crystallographic model of human Ctr1^18,19^, with each subunit containing an extracellular N-terminal region followed by three transmembrane helices (TM1-3) and an intracellular C-terminus (Fig. 1c, d). To validate that sCtr1_cryst_, with an insertion of the crystallization chaperone BRIL in the TM1–TM2 loop, retains native conformation, we placed the sCtr1_cryst_ trimer into the electron crystallographic reconstruction map of hCtr1 (EMD-1593, Fig. 2), and demonstrated that indeed the transmembrane helices of sCtr1_cryst_ align well with those of the full-length human Ctr1 protein. This observation indicates that the overall structures are conserved between the human and fish orthologs and that the crystallization chaperone BRIL did not substantially alter the native conformation of sCtr1. Therefore, our structural and functional analyses using the engineered sCtr1_cryst_ construct are generally applicable to the Ctr1 family of Cu transporters.

**Fig. 2:**
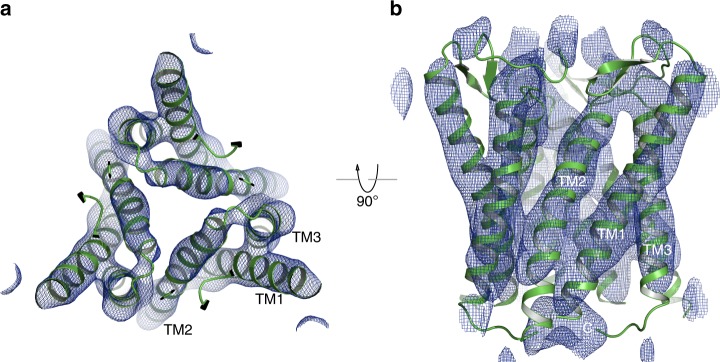
Comparison of X-ray structure of sCtr1_cryst_ with EM reconstruction of hCtr1. **a** The sCtr1_cryst_ trimer structure (in green) is fitted into the electron crystallographic reconstruction of hCtr1 at ~7 Å resolution (EMD-1593), shown as blue mesh. **b** Orthogonal view as in (**a**)

TM1 and TM2 are tilted in the membrane so that TM2 defines the narrow region of the pore near the extracellular side, while TM1 determines the narrow region near the intracellular side of the pore (Fig. 1c, d). TM3 runs approximately perpendicular to the membrane and contacts TM2 from an adjacent subunit. This contact, encompassing a conserved GX_3_G sequence, forms an important assembly interface between subunits as demonstrated by mutagenesis experiments^25^. Structurally, the GX_3_G sequence in TM3 is located at a helix crossover region where TM1 from the same subunit and TM2 from an adjacent subunit cross each other (Supplementary Fig. 5). Tight packing interactions in this region are enabled by glycine residues, which have the smallest side chains, whereas bulky side chains at these positions would impair the intimate assembly interface and hence undermine Ctr1 function. Notably, the GX_3_G sequence in Ctr1 extends to a glycine zipper motif (G/S/AX_3_GX_3_G) (Supplementary Fig. 1), a common structural motif found in many ion channels that is critical for channel function^26^.

### Ion conduction pathway

The sCtr1 structure shows that the potential Cu^+^ ion conduction pore aligns with the central three-fold symmetry axis between identical subunits, reminiscent of the classical architecture of many ion channels such as the tetrameric cation channel superfamily^27^. The structure, together with energy-independent Cu^+^ transport across membranes^4^, suggests that Ctr1 could in principle function like an ion channel. However, the conduction rate for Cu^+^ ions was estimated to be ~10 ions per second per trimer in cultured cells for human Ctr1^28^. Notably, in vitro Ag^+^ transport experiments using a thermophilic fungal Ctr1 homolog corroborated a similarly slow rate^29^. This rate is orders of magnitude lower than the rates of most characterized ion channels and confirms a moderate rate of transport^30^. A molecular understanding of this unique Cu^+^ passage through Ctr1 has remained a mystery.

**Fig. 3:**
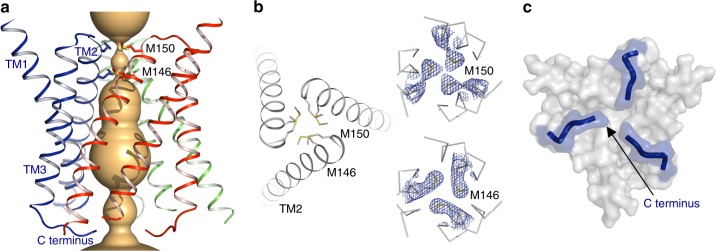
Ion conduction pore. **a** The central ion conduction pore of sCtr1, shown as orange surface. **b** The Cu^+^ selectivity filter. Two methionine rings (M146 and M150 in TM2) close to the extracellular side form the selectivity filter (left panel). The refined 2F_o_−F_c_ electron density for the methionine side chains (contoured at 1.7 σ) is shown as blue meshes (right panels). **c** A hypothetical intracellular gate. Shown is the surface representation of sCtr1 from the intracellular side. The C-terminal tail following TM3 extends to the central pore axis, potentially forming an intracellular gate

To investigate potential Cu^+^ ion permeation mechanisms, we calculated the pore dimensions along the central ion conduction pathway^31^ (Fig. 3a). Essential for Cu^+^ uptake activity^20,32^, two strictly conserved methionine residues in an MX_3_M sequence motif in TM2 (M146 and M150 in sCtr1) form two layers of methionine triads, constituting a potential Cu^+^ selectivity filter at the extracellular entrance (Fig. 3a, b). With two Cu^+^-binding sites within the filter, the side-chain sulfur atoms from each triad generate each Cu^+^ binding site. The ion conduction pore widens below the selectivity filter owing to tilting of the pore-lining helices TM2, resulting in an enlarged central cavity (Fig. 3a). Meanwhile, TM1 tilts towards the central pore axis and lines the pore close to the intracellular side (Fig. 3a), suggesting its role in compensating further TM2 opening and thereby restricting the pore diameter. Notably, these narrow dimensions at the methionine triads would preclude the proposed permeation of larger cisplatin (Pt(NH_3_)_2_Cl_2_) molecules through Ctr1 and would, therefore, favor an alternative mode of import such as cisplatin-induced Ctr1 endocytosis^33^.

Following TM3, the intracellular C-terminus harbors a highly conserved HCH motif that forms an additional Cu^+^-binding site, potentially relaying Cu^+^ to cytoplasmic Cu chaperones^34^. In the crystal structure, the C-terminal tail extends toward the central pore axis (Fig. 3c). Interestingly, the last four amino acids in the C-terminus, including the HCH motif, are the only segment of sCtr1_cryst_ not resolved in the electron density map, implying a structurally flexible C-terminal tail. Conceptually, the C-terminal tail could extend to the central pore and form an intracellular gate to seal the pore, reminiscent of a gating behavior referred to ‘ball-and-chain’ inactivation in ion channels^35^. Displacement of the cytosolic C-terminal tail from the central pore would open the gate and allow the peptide to transfer bound Cu^+^ to cytoplasmic chaperones or other ligands^34^. The possibility that the Ctr1 C-termini function as an intracellular gate is supported by three additional lines of evidence. First, fluorescence resonance transfer (FRET) experiments demonstrate that the C-termini move during Cu transport^17^. Second, C-terminal truncations of Ctr1 exhibit substantially elevated transport rates in cell culture^28^. Third, placement of the sCtr1_cryst_ structure into the electron crystallographic reconstruction of hCtr1 indicates that the C-termini extend into strong electron density along the central three-fold symmetry axis, perhaps representing the intracellular gate (Fig. 2b).

### Cu^+^ binding and permeation

**Fig. 4:**
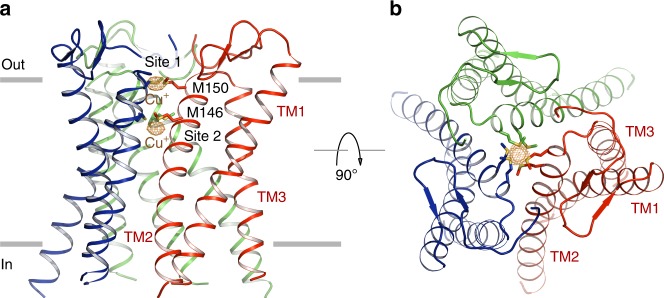
Cu^+^ selectivity filter. **a** Two bound Cu^+^ ions at the selectivity filter. The anomalous difference electron density, calculated from diffraction data in the 20–3.6 Å resolution and contoured at 8.0 σ, is shown as orange mesh, indicating bound Cu^+^ ions. Also shown are the side chains of the methionine residues (M146 and M150) in the filter. **b** Orthogonal view as in (**a**)

To further understand the structural basis for ion selectivity, we determined the Cu^+^-bound sCtr1_cryst_ structure at 3.2 Å resolution (Table 1). By measuring X-ray diffraction at a wavelength near the Cu absorption edge, we calculated the anomalous difference electron density map and unambiguously located two bound Cu^+^ ions along the central three-fold pore axis in the selectivity filter (Fig. 4a, b), consistent with a previous X-ray absorption spectroscopy study demonstrating that the human Ctr1 trimer stably binds two Cu^+^ ions through 3-coordinate Cu-S bonds^19^. Each of the two methionine triads indeed binds a single Cu^+^ ion through Cu-S coordination, with an ~8 Å distance between the two bound Cu^+^ ions at sites 1 and 2. As observed in our structure, Cu^+^ coordination by three Cu-S bonds, which is commonly employed by Cu^+^-handling proteins including a prokaryotic Cu^+^ efflux pump CusA^19,36,37^, likely confers ion selectivity against Cu^2+^ and other transition metal ions. The trigonal planar geometry provided by the three thioether groups of methionine for Cu^+^-coordination is distinct from the coordination geometry for Cu^2+^, which requires four or more ligands^38^. The two methionine triads in the selectivity filter are both essential for Cu^+^ conduction; mutation of either methionine to leucine (M146L or M150L) and the double methionine mutant (M2L) abolish Cu transport activity, as demonstrated by radioactive ^64^Cu uptake and yeast complementation assays (Fig. 1a, b). Owing to the limited resolution of the sCtr1_cryst_-Cu^+^ structure, the methionine side chains cannot be precisely modeled in the selectivity filter and therefore the geometry and distance of Cu-S coordination cannot be accurately determined. Further studies with atomic-resolution structures are required to define the Cu-S coordination chemistry in the Ctr1 selectivity filter.

The Cu^+^-free and Cu^+^-bound sCtr1_cryst_ structures are essentially identical with an r.m.s.d (root-mean-square deviation) of ~0.2 Å for all Cα atoms, indicating that no significant conformational changes are associated with permeant Cu^+^ ion binding within the structured regions in the crystal structures of sCtr1_cryst_. The invariant selectivity filter conformation, albeit at medium resolution, suggests a fixed pore configuration for selective Cu^+^ permeation. However, one noticeable difference is that, in the Cu^+^-bound structure, the C-terminus following TM3 is more disordered, with four additional amino acids absent from the electron density map. The lack of resolved electron density for this region might be owing to the lower resolution of the Cu^+^-bound structure. Nonetheless, this observation aligns with the suggestion that in addition to a role in donating Cu^+^ to intracellular Cu^+^ chaperones^34^, the Ctr1 C-terminal tail might function as a gate, likely adopting a more dynamic conformation during Cu^+^ conduction. Interestingly, the dynamic nature of Ctr1 during Cu^+^ transport is supported by FRET analysis of yeast Ctr1 and the Cu-stimulated endocytosis and degradation of human Ctr1^17,39^. However, the speculation of the C-termini operating as a gate certainly requires further studies.

**Fig. 5:**
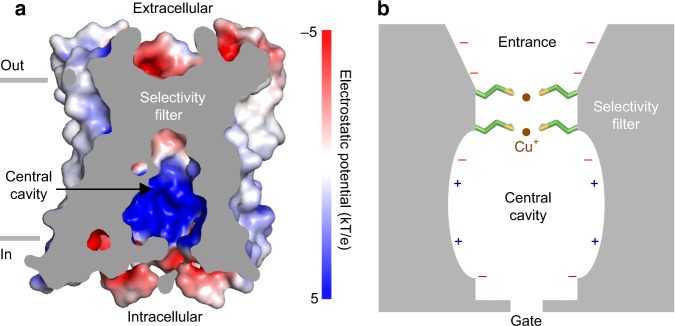
Cu^+^ permeation. **a** Cross-section view showing surface of the sCtr1 ion conduction pore colored by electrostatic potential (red, −5 kT/e; white, neutral; blue, +5 kT/e), with the relationship of the pore relative to the cell membrane shown. **b** Proposed mechanism of Ctr1 Cu^+^ ion selectivity and permeation. Only two subunits are shown for clarity. Two methionine rings coordinate two Cu^+^ ions. Electrostatic potential along the ion pore is indicated

We analyzed sCtr1_cryst_ surface electrostatic potential to gain insights into the Cu^+^ permeation mechanism. Polar amino acids line the central pore and thus likely facilitate charge movement across otherwise hydrophobic membranes (Fig. 5 and Supplementary Fig. 6). Negative electrostatic potential at the extracellular entrance above the methionine-rich selectivity filter would attract positively charged Cu^+^ ions. Preceding the extracellular entrance, Ctr1 harbors an N-terminal metal-binding domain (MBD), which can bind multiple Cu^+^ ions^40–44^. Although the N-terminal MBD is not required for Cu transport activity, mutants lacking the MBD display slower rates of transport^45–47^. Consistently, in the ^64^Cu uptake experiments, sCtr1 with the deletion of the MBD showed a reduced transport rate (Supplementary Fig. 3). Therefore, the N-terminal MBD potentially increases the local Cu^+^ concentration and enhances Cu^+^ access to the pore from the extracellular side^40,48^.

### Zinc binding site

**Fig. 6:**
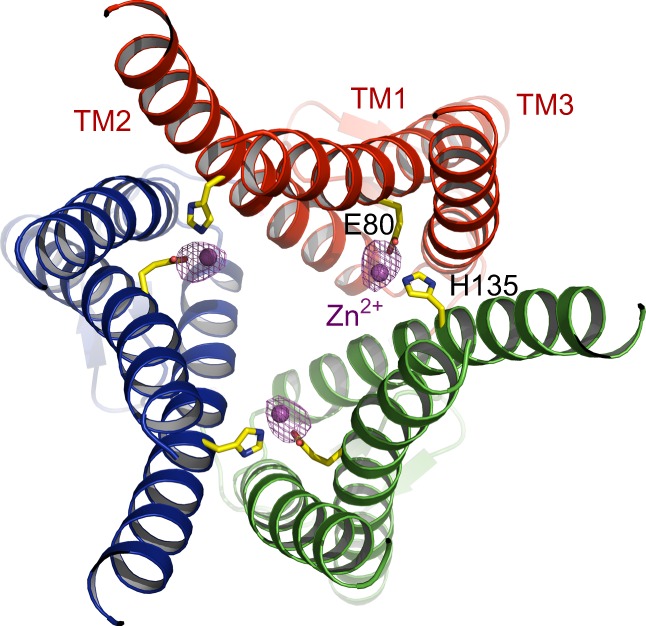
Zn-binding site. sCtr1_cryst_ is viewed from the intracellular side. Zn^2+^ ions are shown as magenta spheres and the anomalous difference electron density contoured at 6.0 σ is shown as magenta mesh

Crystals of sCtr1_cryst_ appeared from multiple crystallization conditions. However, only crystals grown in crystallization conditions containing zinc acetate diffracted X-rays well and allowed structure determination. Coincidently, zinc acetate is also a therapeutic agent for the treatment of Wilson’s disease, a malady that results from hepatic Cu overload and toxicity^49^. We, therefore, reasoned that Zn^2+^ ions might bind to sCtr1_cryst_, stabilizing the structure. To test this hypothesis, we measured X-ray diffraction of Cu^+^-free crystals near the Zn absorption edge (Table 1) and the anomalous difference peaks unambiguously identified a Zn^2+^ binding site comprised of E80 in TM1 and H135 in TM2 from an adjacent subunit (Fig. 6). Interestingly, amino acid substitutions at the two analogous positions in human Ctr1 (E84Q and H139R) substantially increase the Cu^+^ transport rate in cell culture^32^. Consistently, the corresponding mutants in sCtr1 (E80Q and H135R) showed increased Cu uptake rates (Supplementary Fig. 3b). Moreover, the corresponding H139R mutation in human Ctr1 decreases Cu-dependent endocytosis^28^, suggesting that Zn^2+^ binding might be required for Cu-induced endocytosis. The Ctr1 structure, together with the mutagenesis analysis, suggests that Zn^2+^-binding could negatively regulate Cu^+^ transport activity and might contribute to the mechanism by which zinc dampens intestinal Cu absorption in patients with Wilson’s disease. However, the concentration of zinc acetate in crystallization conditions (50 mM) appears to be out of the range of physiological Zn^2+^ concentrations and therefore the physiological relevance of Zn^2+^-binding requires further structural and biochemical studies.

## Discussion

Despite the critical importance of Cu in health and disease, we have only a rudimentary understanding of the molecular basis of Cu transport across biological membranes, owing to a lack of high-resolution three-dimensional structures and relevant biochemical and biophysical characterization. In this study, we present crystal structures of a metazoan Ctr1 protein in the Cu^+^-free and Cu^+^-bound states, establishing a molecular framework for understanding ion selectivity and permeation for this essential element. Cu^+^ transported by Ctr1 appears to engage an ion channel-like mechanism, with two critical methionine rings potentially constituting a Cu^+^ selectivity filter near the extracellular entrance, the substitution of which abolishes Cu^+^ transport activity^48^. Permeation of Cu^+^ through the membrane is likely facilitated and regulated by the electric field that entering ions experience in the central pore formed by the Ctr1 homotrimer. Exit of Cu^+^ ions from the selectivity filter is promoted by negative electrostatic potential within the cavity, immediately below the filter. The central cavity, with positive electrostatic potential, likely presents an energy barrier limiting the Cu^+^ ion conduction rate, which may explain the observed slow conduction rate for an essential but potentially toxic ion^28^ (Fig. 5b). On the other hand, since the central cavity is wide, anions may shield positive potential and reduce the energy barrier for Cu^+^ movement. Direct measurements of the rates of Cu^+^ conduction in a reconstituted system for the wild type and mutants of the charged residues in the central cavity would help address these possibilities.

Interestingly, the selectivity filter of Ctr1 is comprised of methionine residues that typically would form a hydrophobic seal in other ion channels. The thioether group from methionine is a common ligand for Cu^+^ but not for other cations such as Na^+^, K^+^, and Ca^2+^. This discrimination might be a reason why other ion channels often utilize the hydrophobic nature of methionine for channel gating while Ctr1 exploits the thioether group for selective Cu^+^ binding. Intriguingly, the Ctr1 methionine filter might serve both purposes by providing selectivity and limiting the flow rate. However, it remains to be determined whether the Cu^+^ selectivity filter can be tuned for conduction of other metal ions through the Ctr1 pore, by introducing mutations of these two methionine residues constituting the selectivity filter.

Although the Cu^+^-bound and Cu^+^-free sCtr1 structures show striking similarity, it is interesting to note that a larger portion of the C-terminal tail is not resolved in the Cu^+^-bound structure. Previous studies have shown that deletions in the C-terminal tail abolish Cu-mediated endocytosis^28^, thereby increasing cellular Cu^+^ uptake. Additionally, H139 in human Ctr1, corresponding to H135 bound to Zn^2+^ in the sCtr1 crystal structures, functions in Cu-mediated endocytosis^32^. Importantly, combining deletion of the C-terminal tail with mutation of H139 is not additive with respect to Ctr1 activity, suggesting that these residues contribute to a similar function^32^. Collectively, these results point to an intriguing possibility that Cu^+^ bound to the selectivity filter Met residues can induce structural alterations through the Zn^2+^-binding residues and eventually to the C-terminal tail, which stimulates endocytosis to prevent prolonged Cu^+^ uptake and eventual toxicity. Furthermore, together with electron crystallography and mutagenesis studies^17,19,28,34^, our structures also suggest that the cytoplasmic C-terminal tail could act as a potential dual-functional flexible intracellular gate that regulates ion passage and transfers transported Cu^+^ ions to cytoplasmic chaperones. Undoubtedly, mechanistic insights into Cu^+^ coordination chemistry, Cu^+^ handoff to intracellular chelating agents, Zn^2+^ modulation of transport activity, and the potential roles of the cytosolic C-terminus await further structural and biochemical analyses. These avenues will facilitate the development of mechanism-based therapeutics for the treatment of numerous pathological conditions associated with disturbed Cu metabolism such as hepatic Cu accumulation that underlies Wilson’s disease.

## Methods

### Cloning and expression and purification

The gene encoding *Salmo salar* Ctr1 (Gene ID: 100380822) was codon optimized, synthesized (GENEWIZ), and subcloned into a modified *Pichia pastoris* expression vector pPICZ-B (Invitrogen) using XhoI and EcoRI restriction sites. The expressed protein contains a C-terminal PreScission protease cleavage site followed by a GFP-His_10_ tag. This plasmid construction results in the addition of a stretch of amino acids (SNSLEVLFQ) to the C-terminus of the expressed protein after protease cleavage. The full-length construct yielded crystals that diffracted X-rays only to ~8 Å resolution. For structure determination, we identified a functional construct sCtr1_cryst_, in which the N-terminal 40 residues were deleted and an intracellular loop (residues 94–120) was replaced by a fusion protein, the thermostabilized apocytochrome BRIL^23^.

*Pichia pastoris* strain SMD1163H (Invitrogen) was used for large-scale protein expression. Cells were disrupted by milling (Retsch MM400), and re-suspended in buffer containing 50 mM Tris pH 8.0, 150 mM NaCl, 3 μg/ml aprotinin, 2 mM benzamidine, 5 μg/ml leupeptin, 2 μg/ml pepstatin A, 200 μg/ml 4-(2-aminoethyl) benzenesulfonyl fluoride hydrochloride, and 250 μM phenylmethane sulphonylfluoride. The Ctr1 protein was extracted with 2% (w/v) n-dodecyl-β-D-maltopyranoside (DDM, Anatrace) for 3 h at 4 °C. The mixture was centrifuged at 30,000 g for 1 h at 4 °C. The supernatant was incubated with cobalt resin (Clontech) for 3 h at 4 °C with gentle agitation. The resin was then collected and washed with 10 bed volumes of buffer containing 20 mM Tris pH 8.0, 150 mM NaCl, 30 mM imidazole, and 4 mM DDM. The C-terminal GFP tag was removed by incubation of the resin with PreScission protease overnight at 4 °C. The Ctr1 protein was then collected, concentrated and further purified on a Superose 6 gel-filtration column (GE Healthcare) equilibrated with buffer containing 20 mM Tris pH 8.0, 150 mM NaCl, 5 mM dithiothreitol (DTT), 5 mM 5-Cyclohexyl-1-Pentyl-β-D-Maltoside (CYMAL-5, Anatrace). Peak fractions were concentrated to ~7 mg/ml for crystallization experiments.

For crosslinking experiments, sCtr1_cryst_ was purified as described above except for the gel-filtration buffer, which contains 20 mM HEPES pH 7.5, 150 mM NaCl, and 5 mM CYMAL-5. Protein was then concentrated to ~1.0 mg/ml and treated with disuccinimidyl suberate (DSS, ThermoFisher Scientific) ranging from 0 to 700 μM at room temperature for 1 h. The reactions were quenched by addition of 70 mM Tris pH 8.5 and analyzed by SDS-PAGE.

### Crystallization and structure determination

Crystals were grown by hanging-drop vapor diffusion at 20 °C. Crystals appeared in 2 days and grew to full size within 2 weeks in crystallization condition containing 26% PEG 400, 50 mM zinc acetate, and 50 mM sodium cacodylate pH 5.9. Tantalum-derived crystals were prepared by adding ~0.5 mM tantalum bromide cluster (Ta_6_Br_12_, Jena Bioscience) to drops with crystals, and then soaking for 2 days. To obtain Ctr1-Cu^+^ crystals, purified Ctr1 protein was incubated with 1 mM [Cu(CH_3_CN)_4_]PF_6_ (Sigma) and 2 mM tris(2-carboxyethyl)phosphine (Biosynth) on ice for 1 h before crystallization experiments. Ctr1-Cu^+^ crystals grew in 28% PEG 400, 50 mM zinc acetate, and 50 mM sodium cacodylate pH 5.9. For crystal harvesting, the concentration of PEG 400 in reservoir solutions was increased to 30%, and crystals were equilibrated overnight before flash-freezing in liquid nitrogen.

X-ray diffraction data were collected at the Advanced Photon Source beamlines 24-ID-C and 24-ID-E. Datasets for the sCtr1_cryst_-Cu^+^, Ta_6_Br_12_-derivatized Cu^+^-free, and Cu^+^-free sCtr1_cryst_ crystals were measured at the wavelength of 1.3776 Å (Cu absorption edge), 1.2546 Å (Ta absorption edge), and 1.2820 Å (Zn absorption edge), respectively. Diffraction data were processed with the HKL2000 program suite^50^. Experimental phases to 3.4 Å resolution were calculated from a single-wavelength anomalous diffraction experiment with contributions of individual tantalum atoms from a Ta_6_Br_12_-derived crystal using Phenix AutoSol^51^. Experimental electron density after solvent flattening is of good quality for model building. Each asymmetric unit consists of a single subunit of the sCtr1_cryst_ trimer. The model for BRIL (PDB ID: 1M6T) was placed into the corresponding electron density and manually adjusted. For model refinement of Ctr1-Ta_6_Br_12_, the diffraction data were anisotropically corrected to resolution limits of 3.4, 3.5, and 3.0 Å along the reciprocal cell directions a*, b*, and c* respectively, using the diffraction anisotropy server at the University of California, Los Angeles^52^. For Ctr1-Cu^+^, the corrected resolution limits are 3.6, 3.6, and 3.2 Å along a*, b*, and c* respectively. Iterative model building was carried out in COOT^53^, and rounds of refinement were performed with REFMAC^54^. In the Ctr1-Ta_6_Br_12_ structure, K83 was modeled as alanine and the C-terminal four amino acids were not modeled owing to a lack of electron density. Anomalous difference electron density identified two bound Cu^+^ ions in the selectivity filter in the Ctr1-Cu^+^ structure. F45, K83, and M126 were modeled as alanine and the C-terminal eight amino acids were missing in the model because of poor electron density. The Cu^+^-free and Cu^+^-bound structures are nearly identical with an r.m.s.d of ~0.2 Å for all Cα atoms. We used the high-resolution Cu^+^-free structure for most of our analysis because of more defined side chain conformations. Data collection and model refinement statistics are shown in Table 1. Structural illustrations were prepared using PYMOL (www.pymol.org).

### Functional complementation assay

The *S. cerevisiae* strain MPY17 (*ctr1*Δ*ctr3*Δ) lacking both Ctr1 and Ctr3, which is defective in Cu transport, was used for functional complementation^20,24^. Impaired Cu acquisition in MPY17 leads to dysfunction of cytochrome c oxidase, a mitochondrial Cu-dependent enzyme in the respiratory chain. Consequently, MPY17 is unable to grow on nonfermentable carbon sources with Cu limitation such as the YPEG media (1% yeast extract, 2% Bactopeptone, 2% ethanol, 3% glycerol)^20^. Cell growth in YPEG without the supplement of Cu requires a functional Cu transporter constitutively expressed. The wild-type sCtr1 and mutants were subcloned into the p413GPD vector^20^ using the BamHI and EcoRI restriction sites and transformed into the *ctr1*Δ*ctr3*Δ strain MPY17. Cells transformed with plasmids expressing the wild-type or modified sCtr1 constructs with a C-terminal His_6_ tag were grown to exponential phase (OD_600nm_ ~1.0) in minimal dextrose medium lacking histidine. Cells were then plated as drops with 10-fold serial dilutions on selective media YPEG with or without 100 μM CuSO_4_. Plates were incubated for 7 days at 30 °C and then imaged.

### ^64^Cu uptake assay

For the radioactive ^64^Cu uptake experiments, yeast cells (MPY17) expressing the wild-type or mutant Ctr1 genes with a C-terminal His_6_ tag were grown in minimal dextrose media to exponential phase. To initiate uptake, 2 μCi radioactive ^64^Cu (560–660 mCi/μg of ^64^CuCl_2_, Washington University Cyclotron Facility) was added to 1 ml cell culture with 5 μM CuCl_2_ and then incubated for 20 min at room temperature. 10 mM ice-cold EDTA was added to quench the uptake. Cells were harvested using syringe filters and washed three times with quenching buffer (10 mM EDTA, 0.1 M Tris-succinate pH 6.0). For time-dependent measurements, cells were collected at various time points following uptake initiation. The quantity of radioactive ^64^Cu was determined using γ-counter (PerkinElmer, Inc.), and the values were corrected for isotope decay. Parallel experiments were conducted on ice to estimate the amount of cell-surface Cu-binding and subtract from measurements at room temperature to obtain the net amount of Cu uptake. The Cu transport rates were normalized by cell density. To estimate the relative transport activity, the Cu uptake rates were subtracted from that of the Ctr1-independent background, calibrated by protein expression levels indicated by Western blots, and normalized by the wild type.

Owing to the low level of expression of sCtr1 constructs in *S. cerevisiae*, the Western blots were conducted following partial protein purification to enrich the expressed constructs. Cells expressing various sCtr1 constructs were collected at the same amount and then subjected to affinity purification using cobalt resin. Cells were disrupted by milling and re-suspended in buffer containing 50 mM Tris pH 8.0, 150 mM NaCl, 3 μg/ml aprotinin, 2 mM benzamidine, 5 μg/ml leupeptin, 2 μg/ml pepstatin A, 200 μg/ml 4-(2-aminoethyl) benzenesulfonyl fluoride hydrochloride, and 250 μM phenylmethane sulphonylfluoride. The suspension was then centrifuged at 3500 × *g* for 20 min at 4 °C to remove cell debris. Supernatant was centrifuged at 200,000 × *g* for 1 h at 4 °C to collect total membrane. The plasma membrane was isolated using a sucrose gradient as described^55^. Briefly, total membrane was re-suspended with 20 mM Tris pH 8.0, 1.4 M sucrose. The mixture with sucrose gradients (2.5 ml of 2 M sucrose, 4.5 ml of 1.6 M sucrose, 9 ml of total membrane suspension, 9 ml of 1.2 M sucrose, and 4.5 ml of 0.8 M sucrose) was centrifuged at 160,000 × *g* for 3 h in the Beckman SW 32Ti rotor. The plasma membrane was collected at the 1 M sucrose density region, and solubilized with 20 mM Tris pH 8.0, 150 mM NaCl, and 2% DDM for 3 h at 4 °C. Extraction was then centrifuged at 30,000 × *g* for 1 h at 4 °C and supernatant was collected. The plasma membrane protein extraction was monitored by immunoblotting with antibody against Pma1 (Plasma membrane ATPase 1) (Anti-Pma1, Abcam). Plasma membrane protein mixture was then incubated with cobalt resin (Clontech) for 3 h at 4 °C. The resin was collected and washed with 10 bed volumes of buffer containing 20 mM Tris pH 8.0, 150 mM NaCl, 5 mM imidazole, and 2 mM DDM. The wild-type and mutant sCtr1 proteins with a C-terminal His_6_ tag were eluted with 20 mM Tris pH 8.0, 150 mM NaCl, 500 mM imidazole, and 0.5 mM DDM. Expressed sCtr1 proteins were detected by immunoblotting with anti-His_6_ antibody (GenScript). HRP-conjugated anti-mouse IgG (Santa Cruz Biotechnology) was used as the secondary antibody.

### Reporting Summary

Further information on experimental design is available in the Nature Research Reporting Summary linked to this article.

## Supplementary information

Supplementary Information

Reporting Summary

Source Data

## Data Availability

Data supporting the findings of this manuscript are available from the corresponding author upon reasonable request. A reporting summary for this Article is available as a Supplementary Information file. Atomic coordinates and structure factors for the Cu^+^-free and Cu^+^-bound crystal structures of sCtr1_cryst_ have been deposited into the Protein Data Bank with the accession codes 6M97 and 6M98. The source data underlying Fig. 1b and Supplementary Fig. 3 are provided as a Source Data file.
