# Supplementary material for: X-ray structures of the high-affinity copper transporter Ctr1
Source: Nat Commun. 2019 Mar 27;10:1386. doi: 10.1038/s41467-019-09376-7 (PMC6437178; doi:10.1038/s41467-019-09376-7)
Supplement: Supplementary file 1 — Supplementary Information [file 41467_2019_9376_MOESM1_ESM.pdf]

Supplementary Information

X-ray structures of the high-affinity copper transporter Ctr1

Ren et al.

6 Supplementary Figures

1 Supplementary Table

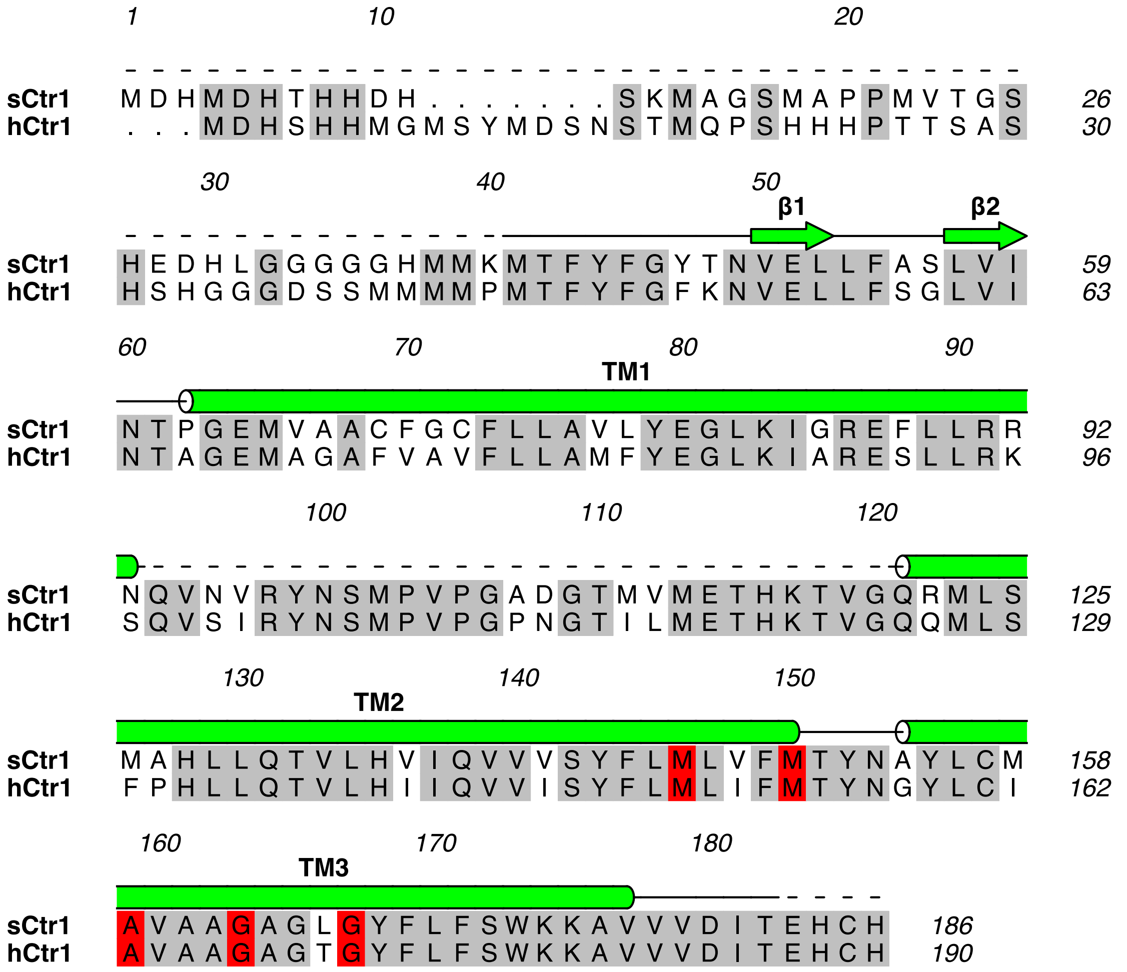

Supplementary Fig. 1 Sequence alignment of salmon and human Ctr1

Secondary structure elements based on the $sCtr1_{cryst}$ structure are shown above the sequence. The conserved 'MX_3M' and '$G/S/AX_3GX_3G$' sequence motifs are highlighted in red. Conserved amino acids are shaded in grey.

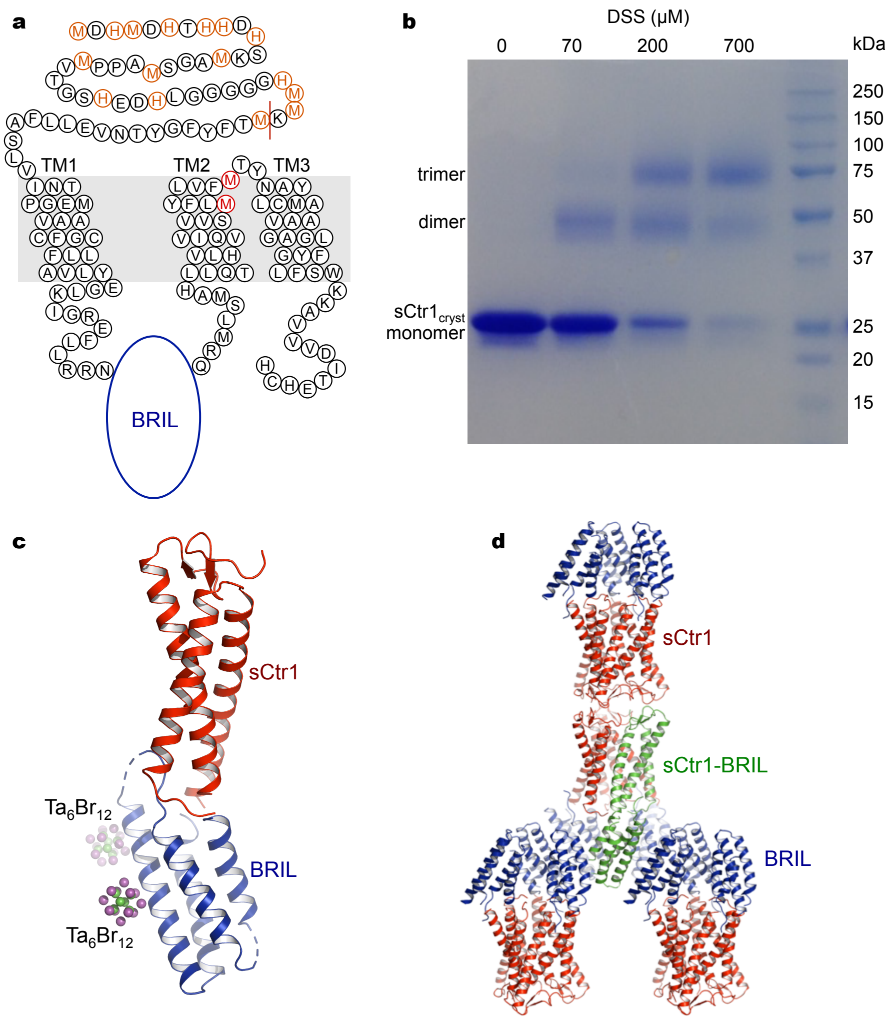

Supplementary Fig. 2 Structure determination of a functional construct sCtr1$_{cryst}$

a, Schematic of the primary sequence of sCtr1$_{cryst}$. The molecular weight of full-length sCtr1 is 21 kDa. The N-terminus is truncated (indicated by a red marker) and a fusion partner BRIL is inserted in an intracellular loop. The Met and His residues potentially involved in Cu^+ binding in the extracellular N-terminal region are highlighted in orange. The critical Met residues required for Cu^+ transport in TM2 are colored in red. **b**, Purified sCtr1$_{cryst}$ protein shows trimeric assembly using crosslinking agent DSS. **c**, Crystal structure of sCtr1$_{cryst}$ withTa_6Br_{12} (green and magenta spheres) bound to the fusion protein BRIL. **d**, Crystal lattice showing important packing interactions mediated by both sCtr1 and BRIL. One of the fusion protomer sCtr1-BRIL in an asymmetric unit is colored in green. The remaining sCtr1 and BRIL molecules in the lattice are colored in red and blue, respectively, to illustrate crystal packing.

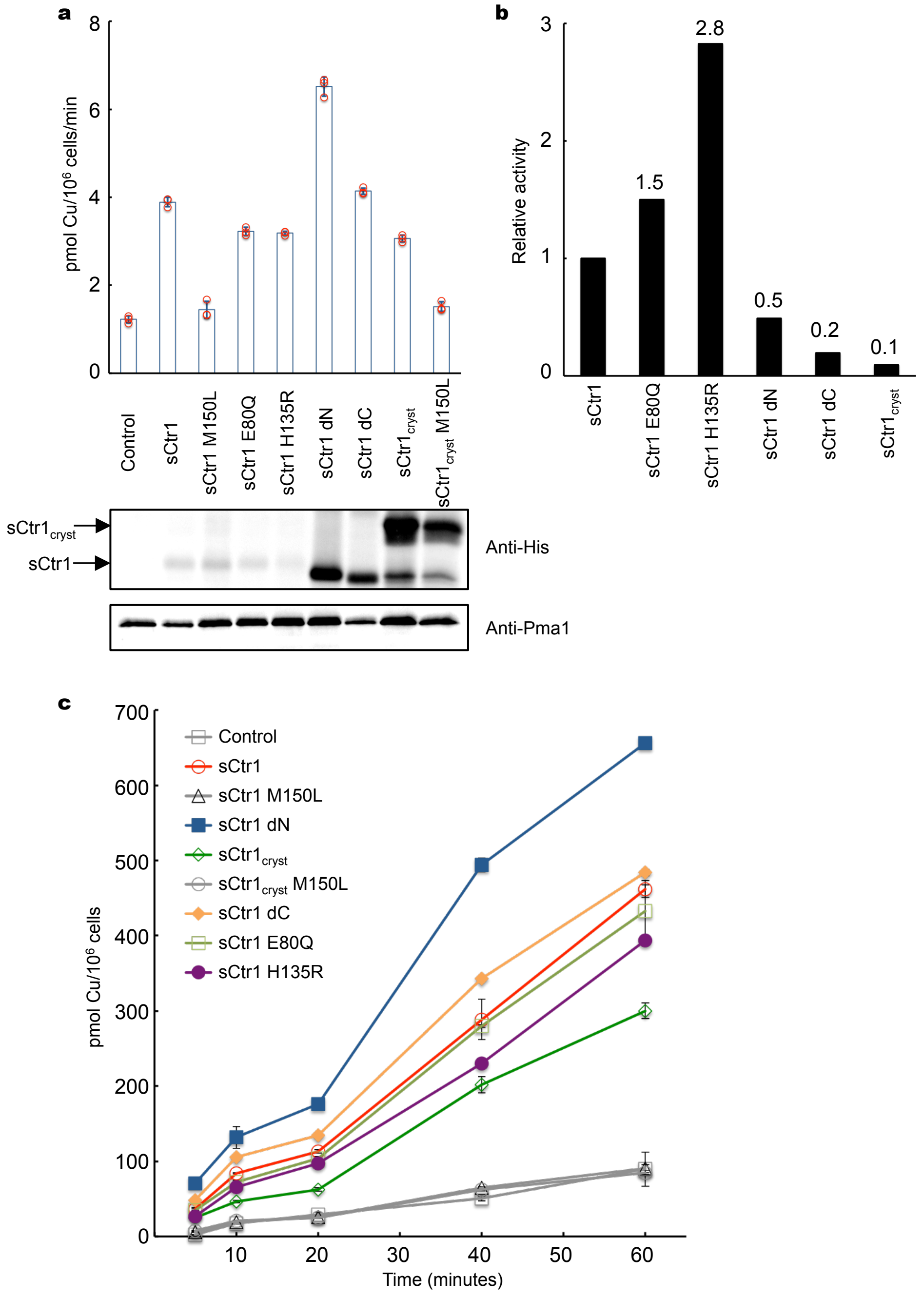

Supplementary Fig. 3 Cu transport activity for wild-type sCtr1 and mutants

a, The upper panel shows ^{64}Cu uptake by the *S. cerevisiae* strain MPY17 expressing constructs including the empty vector (as negative control), wild-type sCtr1, sCtr1 M150L, sCtr1 E80Q, sCtr1 H135R, sCtr1 dN, sCtr1 dC, sCtr1$_{cryst}$, and sCtr1$_{cryst}$ M150L. For sCtr1 dN, the N-terminal 40 residues were removed. For sCtr1 dC, the C-terminal 10 residues including the 'HCH' motif were removed. However, a C-terminal His$_6$-tag was added to all constructs for Western blots. It is therefore important to note that sCtr1 dC does not truly represent sCtr1 with the removal of the C-terminal peptide in that the added His$_6$-tag, potentially interacting with Cu$^+$, might partially restore the function of the

native C-terminal peptide. Values are mean ± s.d. and data were collected from 3 independent measurements. The bottom panel shows Western blots of various sCtr1 constructs expressed in the plasma membrane. **b**, Transport activity for mutants relative to the wild type, normalized by protein expression levels in the plasma membrane. **c**, Time-dependent ^{64}Cu uptake. Values are mean ± s.d. (3 independent measurements). Source data are provided as a Source Data file.

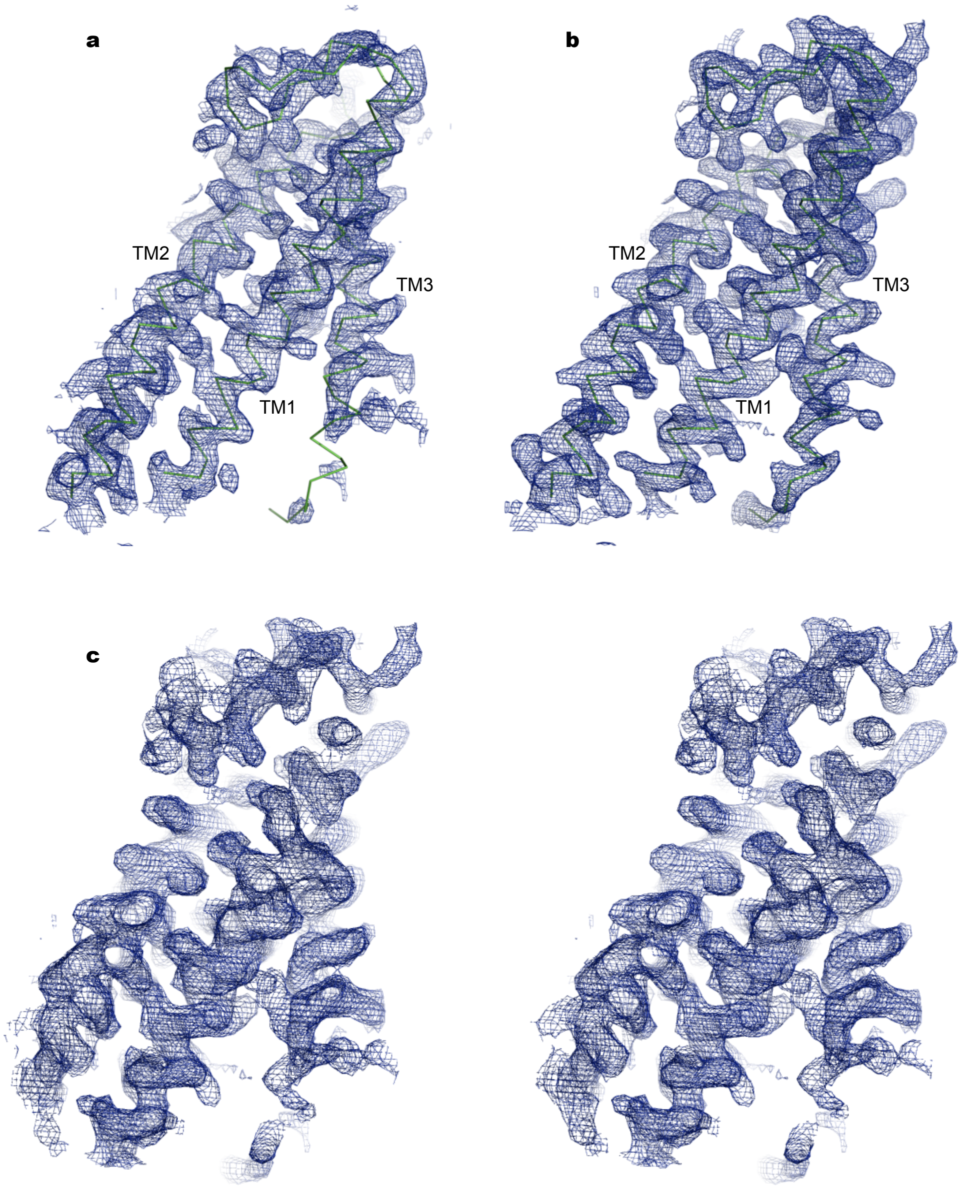

Supplementary Fig. 4 Electron density maps

a, Experimental electron density at 3.4 Å resolution contoured at 1.5 σ. The final refined model is shown in ribbon representation. **b**, Refined $2F_o$-F_c electron density at 3.0 Å resolution contoured at 1.5 σ. **c**, Stereo image of the refined $2F_o$-F_c electron density contoured at 1.5 σ

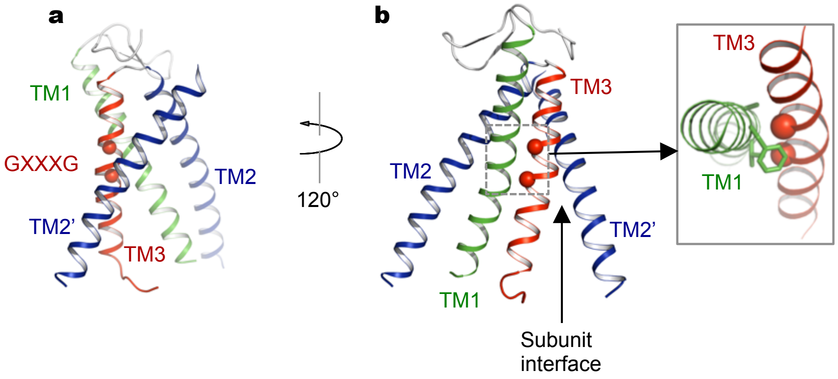

Supplementary Fig. 5 The conserved 'GX_3G' motif is critical for tight helix packing

a and **b,** Two different views showing that the glycine residues (red spheres) are important for tight packing interactions within each subunit (between TM1 and TM3) and between subunits (TM3 and TM2').

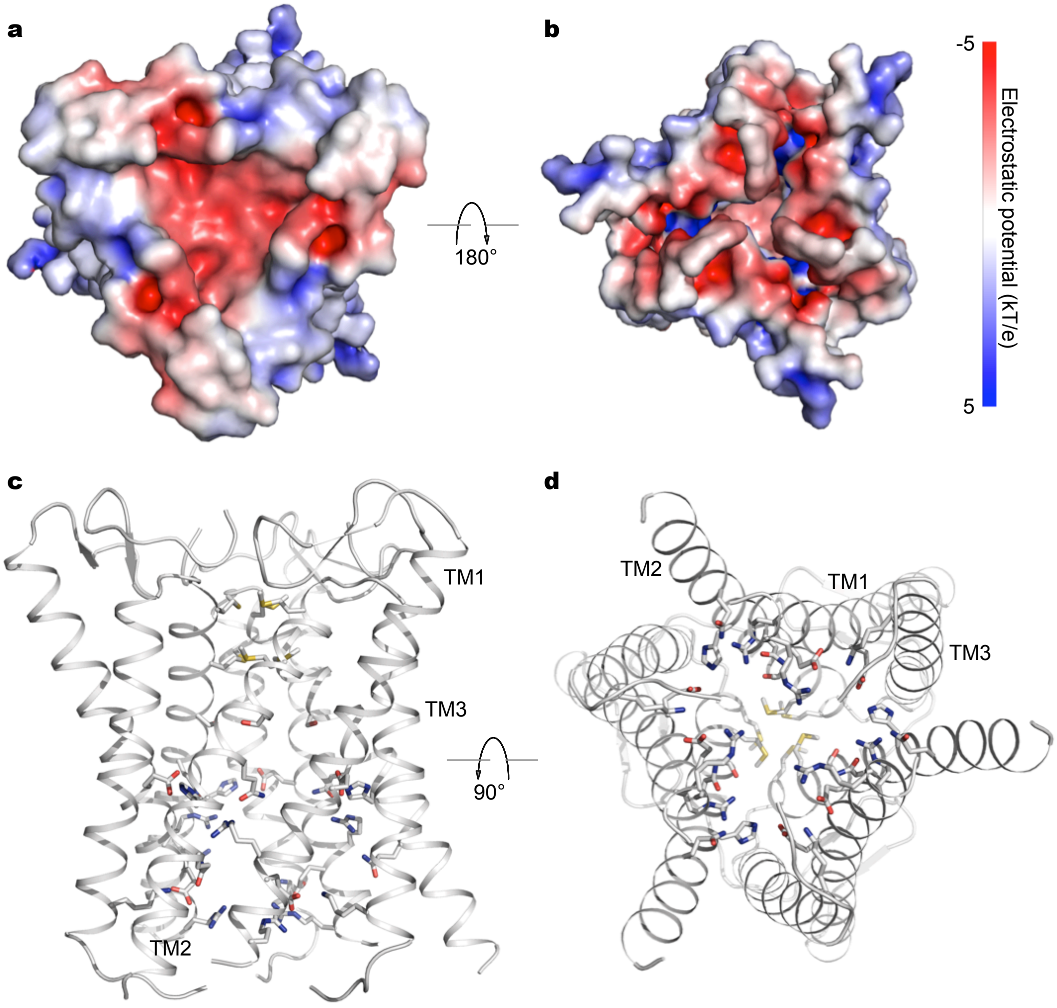

Supplementary Fig. 6 Ion permeation

a, Surface electrostatic potential at the extracellular entrance. **b**, Surface electrostatic potential at the intracellular side. **c** and **d**, Orthogonal views showing polar residues (shown as sticks) lining the ion conduction pore.

Supplementary Table 1 Primers used for cloning

Primer	Sequence
pv1_sCtr1$_{cryst}$_forward1	5'-GCTAGCCTCGAGCCACCATGACCTTCTACTTCGGC-3'
pv1_sCtr1$_{cryst}$_reverse1	5'-GTTGCGACGGAGCAGGAAC-3'
pv1_sCtr1$_{cryst}$_forward2	5'-GTTCCTGCTCCGTCGCAACGCCGACTTGGAAGACAAC-3'
pv1_sCtr1$_{cryst}$_reverse2	5'-CATGCTCAGCATGCGCTGAAGGTATTTTTGGATGTAGGC-3'
pv1_sCtr1$_{cryst}$_forward3	5'-CAGCGCATGCTGAGCATG-3'
pv1_sCtr1$_{cryst}$_reverse3	5'-CAAAACTTCCAAAGAATTCGAGTGGCAGTGCTCGGTGATG-3'
p413GPD_sCtr1_forward<br>p413GPD_sCtr1dC_forward	5'-GTCAGTGGATCCATGGACCACATGGACCAC-3'
p413GPD_sCtr1dN_forward<br>p413GPD_sCtr1$_{cryst}$_forward	5'-GTCAGTGGATCCATGACCTTCTACTTCGGC-3'
p413GPD_sCtr1_reverse<br>p413GPD_sCtr1dN_reverse<br>p413GPD_sCtr1$_{cryst}$_reverse	5'-GTCAGTGAATTCTTAGTGGTGATGGTGATGGTGGTGGCAGTGCTC<br>GGTGATGTC-3'
p413GPD_sCtr1dC_reverse	5'-GTCAGTGAATTCTTAGTGGTGATGGTGATGGTGGGCCTTCTTCCA<br>GCTAAACAG-3'
p413GPD_sCtr1M2L_forward<br>p413GPD_sCtr1$_{cryst}$M2L_forward	5'-CTGCTCGTGTTCCTGACCTACAACGCCTACCTG-3'
p413GPD_sCtr1M146L_forward<br>p413GPD_sCtr1$_{cryst}$M146L_forward	5'-CTGCTCGTGTTCATGACCTACAACGCCTACCTG-3'
p413GPD_sCtr1M150L_forward<br>p413GPD_sCtr1$_{cryst}$M150L_forward	5'-ATGCTCGTGTTCCTGACCTACAACGCCTACCTG-3'
p413GPD_sCtr1M2L_reverse<br>p413GPD_sCtr1$_{cryst}$M2L_reverse<br>p413GPD_sCtr1M146L_reverse<br>p413GPD_sCtr1$_{cryst}$M146L_reverse<br>p413GPD_sCtr1M150L_reverse<br>p413GPD_sCtr1$_{cryst}$M150L_reverse	5'-CAGGAAGTAGGACACCACC-3'
p413GPD_sCtr1E80Q_forward	5'-CAGGGCCTGAAGATCGGCCGC-3'
p413GPD_sCtr1E80Q_reverse	5'-GTAGAGCACGGCCAGCAGG-3'
p413GPD_sCtr1H135R_forward	5'-AGAGTGATCCAGGTGGTGGTGTC-3'
p413GPD_sCtr1H135R_reverse	5'-CAGCACGGTCTGCAGCAGG-3'
